# Supplementary material for: Tau propagation in the brain olfactory circuits is associated with smell perception changes in aging
Source: Nat Commun. 2024 Jun 6;15:4809. doi: 10.1038/s41467-024-48462-3 (PMC11156945; doi:10.1038/s41467-024-48462-3)
Supplement: Supplementary file 1 — Supplementary Material [file 41467_2024_48462_MOESM1_ESM.pdf]

Supplementary Figures

Supplementary Fig 1

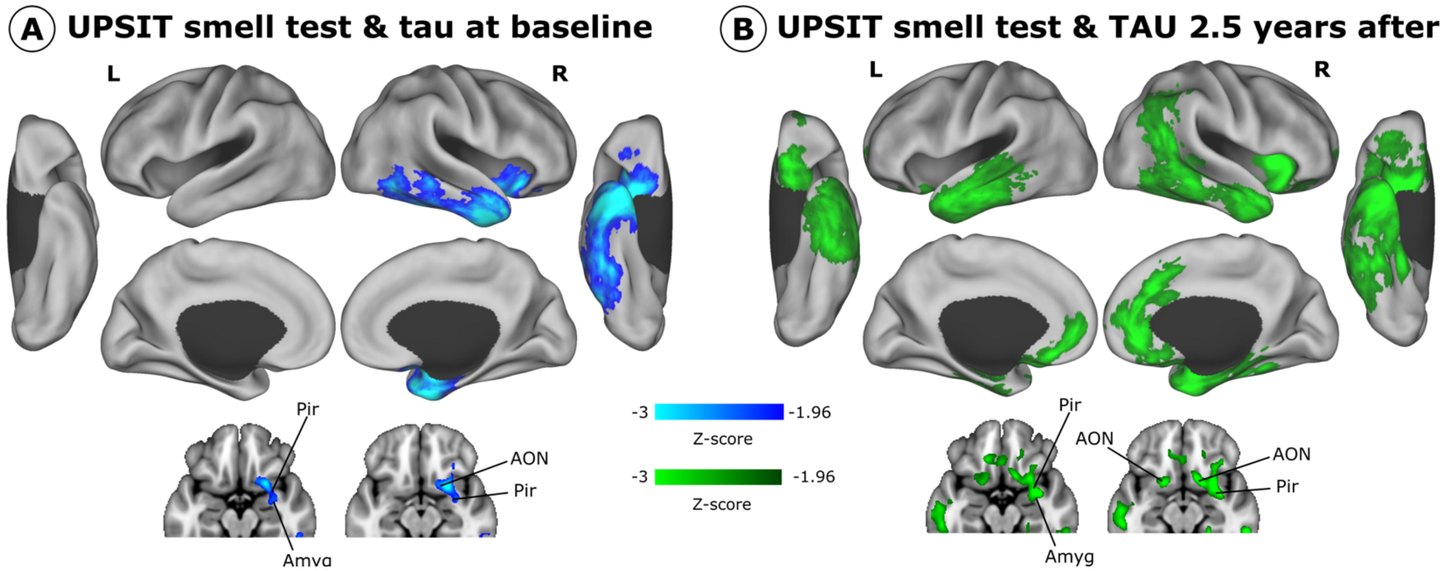

Supplementary Fig. 1 | Smell identification test association with tau accumulation adjusting for age, sex, and smoking history (N=89). A voxel wise general linear model was used to compute the associations. Only results surviving cluster wise multiple comparisons with a p-value<0.05 are displayed. AON - anterior olfactory nucleus, Pir - piriform cortex, Amyg – amygdala.

Supplementary Fig 2

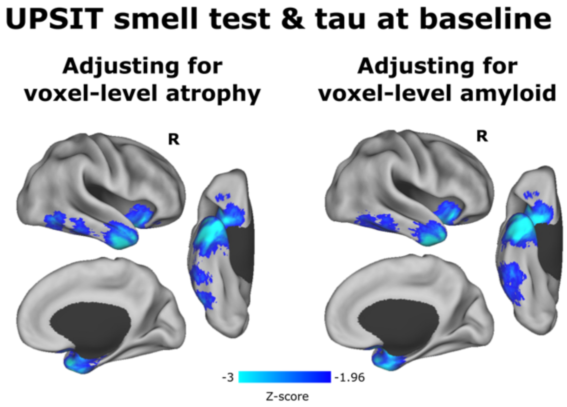

Supplementary Fig. 2 | Smell identification test association with tau accumulation adjusting for atrophy, and amyloid (N=89). A voxel wise general linear model was used to compute the associations. Only results surviving cluster wise multiple with a p-value<0.05 comparisons are displayed.

Supplementary Fig 3

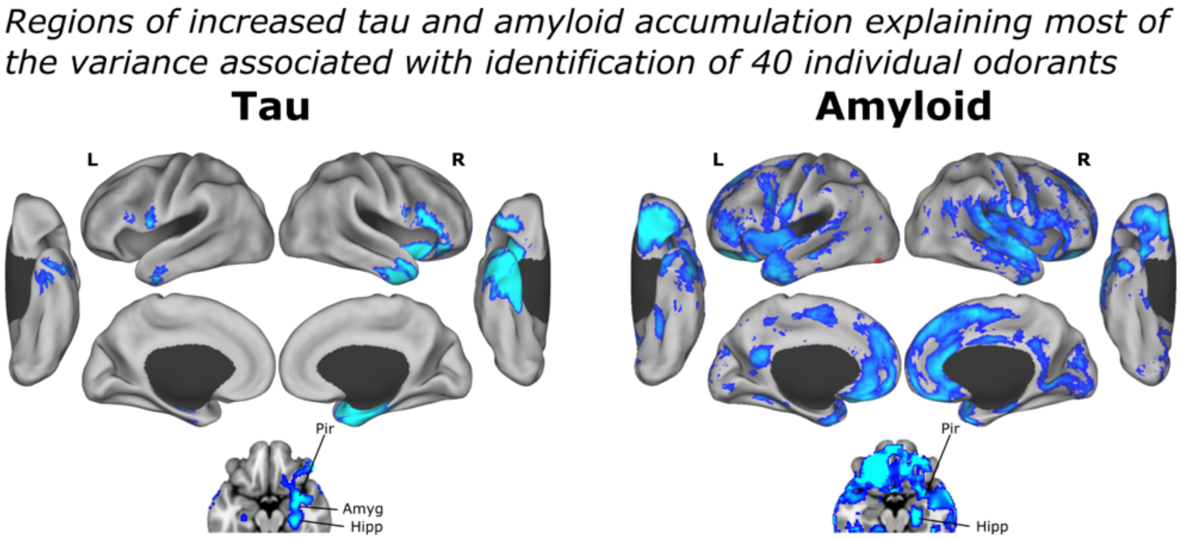

Supplementary Fig. 3 | Voxel-level association between tau and amyloid PET with a linear combination of individual UPSIT items that maximized voxel-level tau and amyloid prediction (N=155). A voxel-wise general linear model was used to compute the association of tau or amyloid accumulation with the linear combination of odorants. Only regions surviving multiple comparisons with a p-value<0.05 are shown.

Supplementary Fig 4

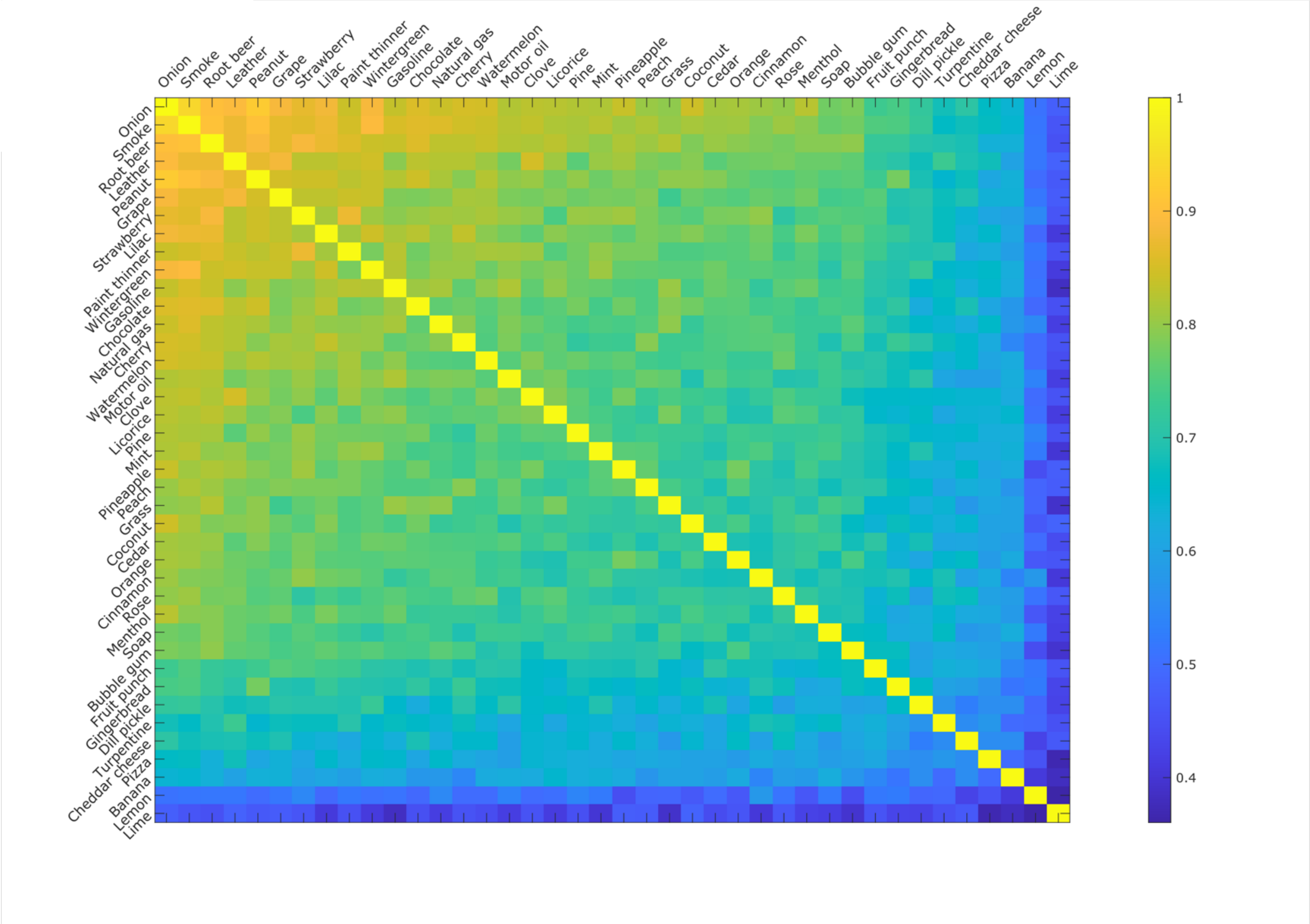

Supplementary Fig. 4 | Similarity (Jaccard index) between the UPSIT items identification ability in N=418.

Supplementary Fig 5

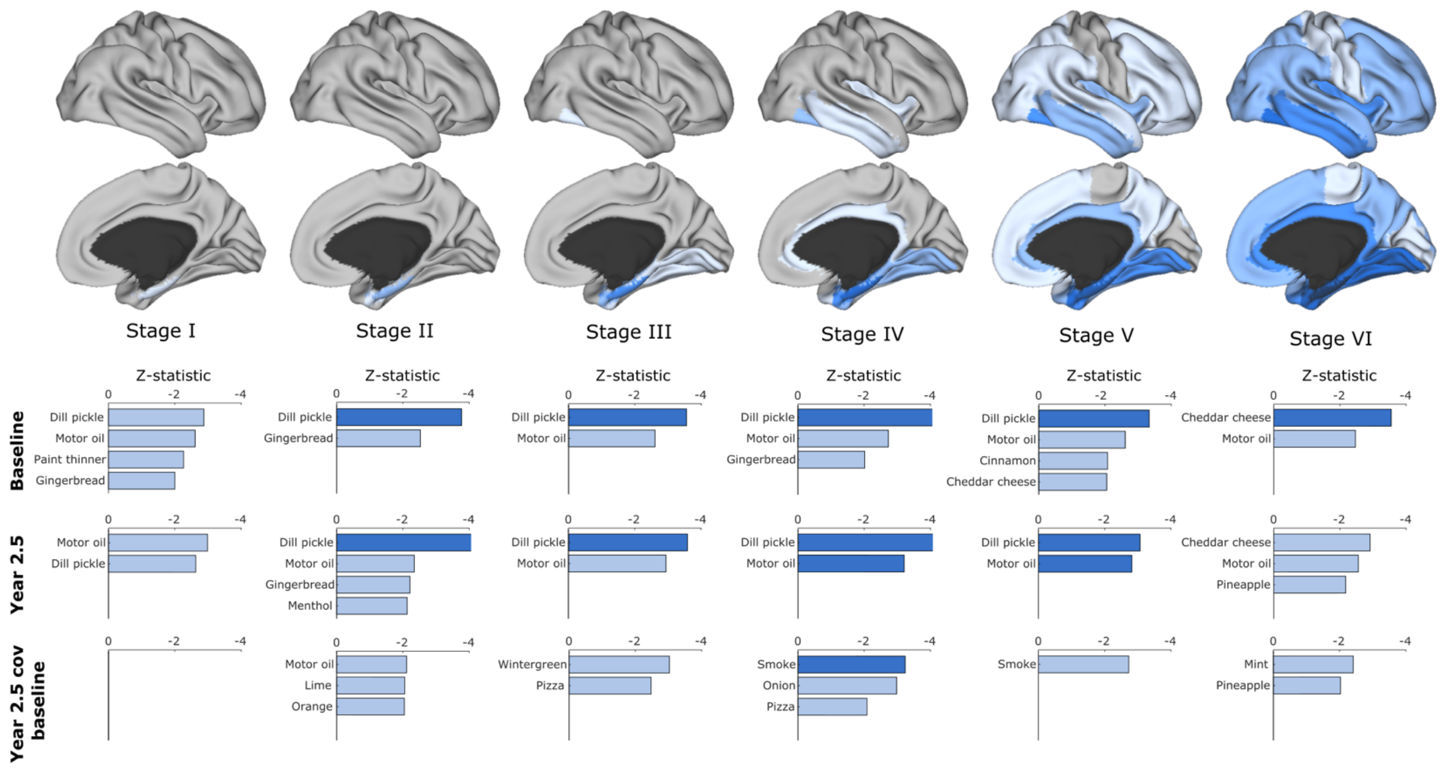

Supplementary Fig. 5 | Odor perception and tau accumulation in Braak stages. Surface projections of brain regions belonging to each Braak stage are displayed. The color intensity represents the amount of tau accumulation expected in the corresponding Braak stage: darker colors show high accumulation. At the bottom of the figure, bar plots represent the z-statistic of the association between each odorant and tau accumulation across the brain regions included in each Braak stage (N=155). A regression model was used to compute the association between tau and each odorant adjusting for age, gender, and smoking history. Only associations with a p-value < 0.05 are displayed, and those surviving the FDR-correction are displayed in darker blue. These associations were computed for baseline odorant measures and both tau at baseline and ~2.5 years after. Additionally, the association of odorant identification and tau ~2.5 years after adjusting for tau at baseline was computed.

Supplementary Fig 6

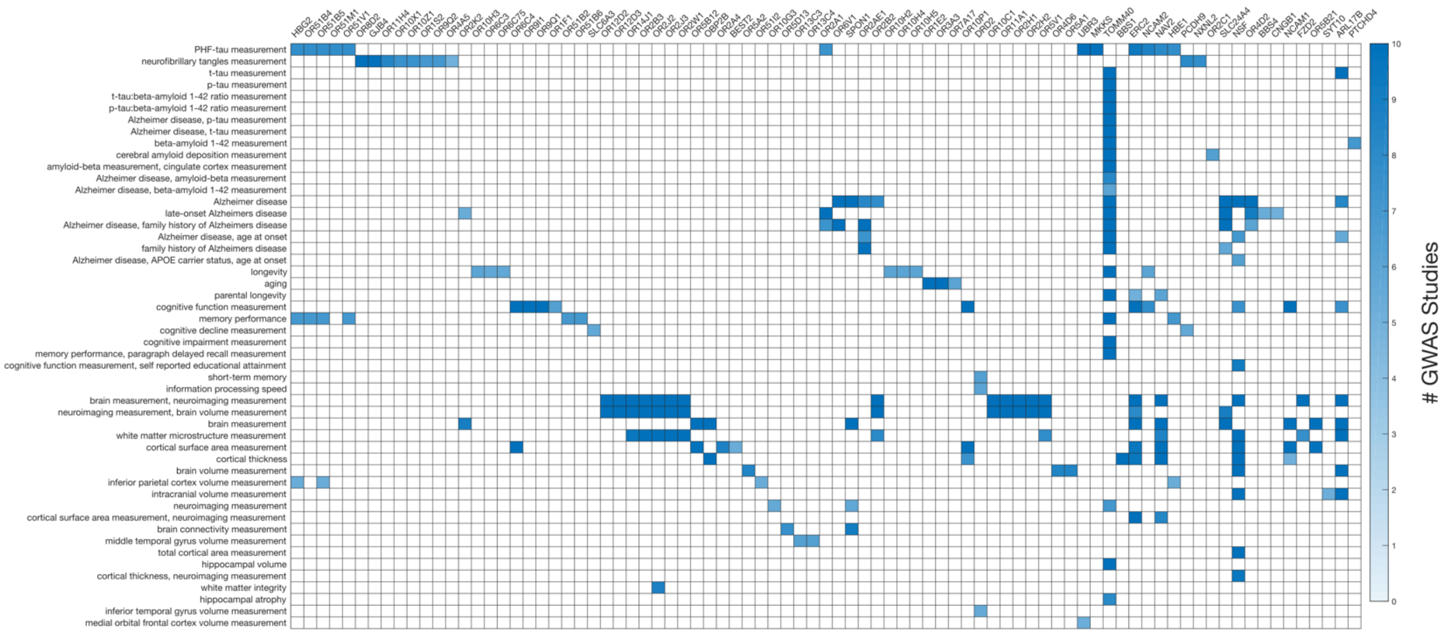

Supplementary Fig. 6 | The GWAS Catalog phenotypes associated with olfactory genes in the Genecard Suite.

Supplementary Tables

Supplementary Table 1. Demographic data for Study Participants (N=418).

Characteristic	Value	Range
Age (years)	72.00 ± 8.34	50 – 91.5
Female/Male	251/167	NA
APOEε4 carrier status, Positive/Negative (NA)	105/261 (52)	NA
UPSIT smell identification test (0-40)	31.81 ± 6.21	7 – 40
Mini-Mental State Examination (0-30)	28.99 ± 1.15	25 – 30
PACC	0.04± 0.72	-2.40 – 2.06

Supplementary Table 2. Demographic data for Study Participants (N=155).

Characteristic	Value	Range
Age (years)	71.05 ± 10.02	50 – 89.75
Female/Male	85/70	NA
APOEε4 carrier status, Positive/Negative (NA)	45/109 (1)	NA
UPSIT smell identification test (0-40)	32.18 ± 6.04	9 – 40
Mini-Mental State Examination (0-30)	29.15 ± 1.10	25 – 30
PACC	0.21± 0.73	-1.94 – 2.35

Supplementary Table 3. Demographic data at baseline for Study Participants (N=89).

Characteristic	Value	Range
Age (years)	73.82 ± 8.44	50 – 89.75
Female/Male	50/39	NA
APOEε4 carrier status, Positive/Negative	30/59	NA
UPSIT smell identification test (0-40)	32.08 ± 5.42	14 – 40
Mini-Mental State Examination (0-30)	29.18 ± 1.12	25 – 30
PACC	0.23± 0.78	-1.94 – 2.35

Supplementary Table 4. Demographic data for Study Participants (N=82).

Characteristic	Value	Range
Age (years)	73.84 ± 8.30	55 – 89.75
Female/Male	48/34	NA
APOEε4 carrier status, Positive/Negative	28/54	NA
UPSIT smell identification test (0-40)	32.35 ± 5.06	14 – 40
Mini-Mental State Examination (0-30)	29.29 ± 1.01	25 – 30
PACC	0.30± 0.72	-1.36 – 2.35

Supplementary Table 5. Associations between tau and total UPSIT score (N=89) in medial temporal and olfactory regions.

	**Cross-sectional**		**Longitudinal**	
	Left	Right	Left	Right
Parahippocampus	T=1:84; p=0.07	**T=2.48; p=0.02**	**T=2.30; p=0.02**	**T=2.67; p<0.01**
Entorhinal	T=1.72; p=0.09	**T=2.54; p=0.01**	**T=2.20; p=0.03**	**T=2.78; p<0.01**
Amygdala	T=1.26; p=0.21	**T=2.27; p=0.03**	T=1.06; p=0.29	**T=2.91; p<0.01**
Hippocampus	**T=2.11; p=0.04**	**T=2.11; p=0.04**	**T=3.11; p<0.01**	**T=2.59; p=0.01**
Piriform	**T=2.26; p=0.03**	**T=2.60; p=0.01**	T=0.65; p=0.51	**T=3.11; p<0.01**
TUB	T=0.62; p=0.54	T=1.56; p=0.12	T=1.85; p=0.06	**T=3.11; p<0.01**
AON	**T=2.69; p<0.01**	**T=2.69; p<0.01**	**T=2.00; p=0.01**	**T=3.18; p<0.01**

Supplementary Methods

Odor Perception and Braak Stages

We also studied whether the ability to identify the 40 odorants of the UPSIT test corresponds with the Braak stages of tau accumulation in the human brain. For all participants with longitudinal data (N=89), we extracted mean bilateral tau intensity values, at baseline and ~2.5 years, of brain regions defining each of the 6 Braak stages. We used a two-sample t-test to compare the tau signal between participants who could identify a particular odor with those who could not – controlling for age, sex, and smoking history – considering each odorant separately (Supplementary Fig5). We observed that specific odor identifications are impaired across Braak stages, such as “motor oil” (Stages I-VI), “dill pickle” (Stages I-V), and “gingerbread” (Stages I, II, VI). By contrast, the loss of other smells is more stage-specific, such as “paint thinner” (Stage I), “menthol” (Stage II), “lime” (Stage II), “orange” (Stage II), “wintergreen” (Stage III), “pizza” (Stage III-IV), “onion” (Stage IV), “smoke” (Stage IV-V), “cheddar cheese” (Stage V-VI), “cinnamon” (Stage V), “pineapple” (Stage VI), and “mint” (Stage VI). Interestingly, “dill pickle” and “motor oil” were the most stable in predicting tau Braak stages at 2.5 years follow-up.

Braak Stages

Braak stage 1 was defined with the perirhinal segmentation of Freesurfer.

The rest of the stages were defined using the Desikan-Killiany atlas.

Braak stage 2: Entorhinal and hippocampus.

Braak stage 3: Parahippocampus, fusiform, lingual, and amygdala.

Braak stage 4: Middle and inferior temporal, caudal, rostral, isthmus, and posterior cingulate and insula.

Braak stage 5: Superior frontal, lateral and medial orbitofrontal, frontal pole, caudal middle frontal, rostral middle frontal, pars opercularis orbitalis and triangularis, lateral occipital, parietal supramarginal, parietal inferior, superior parietal, precuneus, bank superior temporal sulcus and tranvtemp.

Braak stage 6: Pericalcarine, postcentral, cuneus, precentral and paracentral.
