# Supplementary material for: Tau propagation in the brain olfactory circuits is associated with smell perception changes in aging
Source: Nat Commun. 2024 Jun 6;15:4809. doi: 10.1038/s41467-024-48462-3 (PMC11156945; doi:10.1038/s41467-024-48462-3)
Supplement: Supplementary file 3 — Reporting Summary [file 41467_2024_48462_MOESM3_ESM.pdf]

nature portfolio

Corresponding author(s): Jorge Sepulcre

Last updated by author(s): Feb 23, 2024

Reporting Summary

Nature Portfolio wishes to improve the reproducibility of the work that we publish. This form provides structure for consistency and transparency in reporting. For further information on Nature Portfolio policies, see our Editorial Policies and the Editorial Policy Checklist.

Statistics

For all statistical analyses, confirm that the following items are present in the figure legend, table legend, main text, or Methods section.

n/a	Confirmed	
☐	☒	The exact sample size (*n*) for each experimental group/condition, given as a discrete number and unit of measurement
☐	☒	A statement on whether measurements were taken from distinct samples or whether the same sample was measured repeatedly
☐	☒	The statistical test(s) used AND whether they are one- or two-sided<br>*Only common tests should be described solely by name; describe more complex techniques in the Methods section.*
☐	☒	A description of all covariates tested
☐	☒	A description of any assumptions or corrections, such as tests of normality and adjustment for multiple comparisons
☐	☒	A full description of the statistical parameters including central tendency (e.g. means) or other basic estimates (e.g. regression coefficient) AND variation (e.g. standard deviation) or associated estimates of uncertainty (e.g. confidence intervals)
☐	☒	For null hypothesis testing, the test statistic (e.g. *F*, *t*, *r*) with confidence intervals, effect sizes, degrees of freedom and *P* value noted<br>*Give P values as exact values whenever suitable.*
☒	☐	For Bayesian analysis, information on the choice of priors and Markov chain Monte Carlo settings
☐	☒	For hierarchical and complex designs, identification of the appropriate level for tests and full reporting of outcomes
☐	☒	Estimates of effect sizes (e.g. Cohen's *d*, Pearson's *r*), indicating how they were calculated

Our web collection on statistics for biologists contains articles on many of the points above.

Software and code

Policy information about availability of computer code

Data collection	No software was used
Data analysis	FMRIB Software Library v5.0.7 (FSL; https://fsl.fmrib.ox.ac.uk), SPM12 (http://www.fil.ion.ucl.ac.uk/spm), AFNI 20.0.01 (https://afni.nimh.nih.gov/), and FreeSurfer v6 (https://surfer.nmr.mgh.harvard.edu/) were used for image preprocessing. Matlab R2018a (Mathworks Inc., Natick, MA) was used for extended image processing, graph theory and statistical analyses. We used Caret v5 software for imaging surface projections. GeneCards Version 5.11 was used for genetic association analyses.<br><br>All codes related to PET imaging analysis are available for the research community from the corresponding author (J.S.) upon request for the purpose of scientific investigation, teaching or the planning of clinical research studies.

For manuscripts utilizing custom algorithms or software that are central to the research but not yet described in published literature, software must be made available to editors and reviewers. We strongly encourage code deposition in a community repository (e.g. GitHub). See the Nature Portfolio guidelines for submitting code & software for further information.

Data

Policy information about availability of data

All manuscripts must include a data availability statement. This statement should provide the following information, where applicable:

- Accession codes, unique identifiers, or web links for publicly available datasets
- A description of any restrictions on data availability
- For clinical datasets or third party data, please ensure that the statement adheres to our policy

All neuroimaging and clinical data supporting this study's findings are available from https://habs.mgh.harvard.edu/researchers/request-data/public-data-releases. HABS data curation is overseen by Aaron P. Schultz (aschultz@nmr.mgh.harvard.edu) at the Martinos Center for Biomedical Imaging, Massachusetts General Hospital, Harvard Medical School, Boston, MA.

The Allen Human Brain Atlas (AHBA) transcriptomic data is available at https://human.brain-map.org.

Research involving human participants, their data, or biological material

Policy information about studies with human participants or human data. See also policy information about sex, gender (identity/presentation), and sexual orientation and race, ethnicity and racism.

Reporting on sex and gender	All the results were adjusted for self-reported sex information (56.18% were females)
Reporting on race, ethnicity, or other socially relevant groupings	*Please specify the socially constructed or socially relevant categorization variable(s) used in your manuscript and explain why they were used. Please note that such variables should not be used as proxies for other socially constructed/relevant variables (for example, race or ethnicity should not be used as a proxy for socioeconomic status).*<br>*Provide clear definitions of the relevant terms used, how they were provided (by the participants/respondents, the researchers, or third parties), and the method(s) used to classify people into the different categories (e.g. self-report, census or administrative data, social media data, etc.)*<br>*Please provide details about how you controlled for confounding variables in your analyses.*
Population characteristics	Four hundred eighteen cognitive normal participants from the Harvard Aging Brain Study with available smell identification measures were used to model aging effects in olfaction. Eighty-nine of these participants also had longitudinal tau PET imaging. 50 participants (56.18%) were females. At baseline, the mean age of the participants was 73.82 ± 8.44 (SD) and the mean education level was 15.96 ± 2.83 (SD) years. No participants had history of medical or psychiatric disorders and were cognitively unimpaired at baseline: Mini-Mental State Examination (MMSE) > 25 and Clinical Dementia Rating (CDR) = 0. This information is also stated in supplementary tables
Recruitment	Participants were recruited from a longitudinal cohort followed at the Alzheimer Disease Research Center (ADRC) at Massachusetts General Hospital (MGH). In addition, participants were recruited through advertisements in local newspapers, internet sites and community-based outreach events. The Harvard Aging Brain Study has recruited more white and highly educated individuals than expected based on the New England population, therefore results may be less generalizable to other communities.
Ethics oversight	The study complied with all ethical regulations and was approved by the MGB/Partners Human Research Committee at Massachusetts General Hospital. All participants provided written informed consent following the MGB/Partners Human Research Committee regulations, and received monetary compensation after each visit.

Note that full information on the approval of the study protocol must also be provided in the manuscript.

Field-specific reporting

Please select the one below that is the best fit for your research. If you are not sure, read the appropriate sections before making your selection.

[x] Life sciences [ ] Behavioural & social sciences [ ] Ecological, evolutionary & environmental sciences

For a reference copy of the document with all sections, see nature.com/documents/nr-reporting-summary-flat.pdf

Life sciences study design

All studies must disclose on these points even when the disclosure is negative.

Sample size	There is no justification of sample size. This is a longitudinal cohort (panel) study, and this could not be predicted beforehand. All the available participants with longitudinal tau and olfactory measures were used (N=89). However, taking into account previous work in the literature, we were convinced that our sample size would be adequate to achieve the aims of our study. Additionally, using a power analysis the minimun required sample size computed for a 6 parameter multiple regression models with 80% power, 0.05 significance, and Cohen's effect size f2=0.2 is N=67
Data exclusions	No data were excluded. All participants with olfactory measures and longitudinal tau PET were included.

Replication	No formal replication was done, due to the lack of databases with both longitudinal tau pet and olfactory data. A literature search was performed to check if the obtained results resemble the ones obtained by other groups.
Randomization	There is only one experimental group including all the available participants with the required data.
Blinding	All data was acquired as part of the Harvard Aging Brain Study (HABS). The HABS protocol involves blinded subject identifiers for study participants during data sharing and analysis. As our study is based on cognitively normal elderly individuals without any group allocation/ segregation, we believe this point does not apply in our case

Reporting for specific materials, systems and methods

We require information from authors about some types of materials, experimental systems and methods used in many studies. Here, indicate whether each material, system or method listed is relevant to your study. If you are not sure if a list item applies to your research, read the appropriate section before selecting a response.

Materials & experimental systems

n/a	Involved in the study	
☒	☐	Antibodies
☒	☐	Eukaryotic cell lines
☒	☐	Palaeontology and archaeology
☒	☐	Animals and other organisms
☒	☐	Clinical data
☒	☐	Dual use research of concern
☒	☐	Plants

Methods

n/a	Involved in the study	
☒	☐	ChIP-seq
☒	☐	Flow cytometry
☐	☒	MRI-based neuroimaging

Plants

Seed stocks	*Report on the source of all seed stocks or other plant material used. If applicable, state the seed stock centre and catalogue number. If plant specimens were collected from the field, describe the collection location, date and sampling procedures.*
Novel plant genotypes	*Describe the methods by which all novel plant genotypes were produced. This includes those generated by transgenic approaches, gene editing, chemical/radiation-based mutagenesis and hybridization. For transgenic lines, describe the transformation method, the number of independent lines analyzed and the generation upon which experiments were performed. For gene-edited lines, describe the editor used, the endogenous sequence targeted for editing, the targeting guide RNA sequence (if applicable) and how the editor was applied.*
Authentication	*Describe any authentication procedures for each seed stock used or novel genotype generated. Describe any experiments used to assess the effect of a mutation and, where applicable, how potential secondary effects (e.g. second site T-DNA insertions, mosiacism, off-target gene editing) were examined.*

Magnetic resonance imaging

Experimental design

Design type	Voxel based morphometry and diffusion tensor imaging
Design specifications	N/A we do not use fmri task data
Behavioral performance measures	N/A we do not use fmri task data

Acquisition

Imaging type(s)	structural and diffusion
Field strength	3T
Sequence & imaging parameters	The protocol included a T1-weighted magnetization prepared rapid gradient-echo (MPRAGE) scan with the following parameters: repetition time (TR) = 6,400 ms, echo time (TE) = 2.8 ms, flip angle =8°, inversion time (TI) = 900 ms and a voxel size of 1.0 × 1.0 × 1.2 mm. Additionally, a single-shot spin echoplanar sequence was used to acquire diffusion-weighted images with the following parameters: repetition time (TR) = 8,040 ms, echo time (TE) = 84 ms, flip angle = 90°, 2 mm isotropic voxel size, 30 isotropically distributed gradients with a b-value of 700 s/mm2 and 5 non-diffusion weighted images (b = 0 s/mm2)).
Area of acquisition	Whole brain

Diffusion MRI ☒ Used ☐ Not used

Parameters | A single shell 30 isotropically distributed gradients with a b-value of 700 s/mm2 and 5 non-diffusion weighted images were acquired

Preprocessing

Preprocessing software
FMRIB Software Library v5.0.7 (FSL) was used. For vbm, FSLVBM with default parameters was used. For DTI fsl was used with the default parameters for eddy current correction, gradient vector rotation to compensate for head motion and local fitting of the diffusion tensor at each voxel. To model crossing fibers, we used the FSL BEDPOSTX tool with default parameters, and subsequently the PROBTRACKX2 tool taking 1,000 samples from the range of possible principal diffusion directions within each voxel.

Normalization
The structural images were aligned to MNI 152 T1 2mm templates using a non linear registration (FSL fnirt with default parametes). Each individual Fractional anisotropy maps were non-linear coregistered to FMRIB58_FA_1mm template

Normalization template
MNI-152 (2 mm isotropic)

Noise and artifact removal
eddy current correction was used to remove the noise from dti data. Additionally smoothing of the data was also applied to further remove noise

Volume censoring
N/A

Statistical modeling & inference

Model type and settings
A voxel level mass univariate analysis was performed for the associations between olfactory measures and PET and structural neuroimage. A general linear model was used for each voxel adjusting for age, sex, years of education and smoking history. On longitudinal analysis time difference between longitudinal and baseline scan was also included.

For longitudinal spreading of tau we used a bipartite graph between baseline and longitudinal PET data of ROIS of interest (piriform cortex, olfactory tubercle, anterior olfactory nucleus, dorsal raphe nucleus, entorhinal, amygdala, and parahippocampus). For each pair of i,j regions the correlation of participants baseline tau at region i with longitudinal tau at region j was computed controlling for baseline tau at region j . Additionally, to identify the main backbone of tau progression in the olfactory system, we used the skeleton identification step of the PC-algorithm79, an independence-based causal discovery algorithm

Effect(s) tested
N/A

Specify type of analysis: ☐ Whole brain ☐ ROI-based ☒ Both

Anatomical location(s)
Anatomical locations for graph theory approaches were derived from already published anatomical atlases of olfactory regions (see more details in methods).

Statistic type for inference
(See Eklund et al. 2016)
Multiple comparisons correction was applied using Monte Carlo simulation (cluster-wise correction) with 10,000 iterations to estimate the probability of false positive clusters with a p-value of <0.05.

Correction
Multiple comparisons correction was applied using Monte Carlo simulation (cluster-wise correction) with 10,000 iterations to estimate the probability of false positive clusters with a p-value of <0.05.

Models & analysis

n/a	Involved in the study
☒	☐ Functional and/or effective connectivity
☐	☒ Graph analysis
☒	☐ Multivariate modeling or predictive analysis

Graph analysis
A group level connectivity matrix of longitudinal spreading of tau was computed. The connectivity represents the correlation of participants baseline tau at region i with longitudinal tau at region j controlling for baseline tau at region j. Only links with a pvalue<0.05 are shown
